# Pathology

**Published:** 1825-07-01

**Authors:** 


### Pathology.

Actio læsa suum designat singula morbum ;
Præterea morbum partis mutatio signat.

---

1. *Angina Œdematosa.* Some cases of this dangerous disease have been recently published by Dr. Bouillaud, in the Archives, of which we shall take some notice in this place.

*Case* 1. P. Louise, 34 years of age, a cook, of robust habit, was brought to the Hospital Cochin, on the 29th December, in the evening, presenting the following symptoms :—Orthopnœa, inability to open the mouth or to swallow, guttural rattling, vox rauca, sense of suffocation, face pale and slightly bluish, expression of fear and anxiety, eye inanimate and somewhat sunken, prostration of strength, pulse small and rather quick. She stated, though with great difficulty, that she had been ill only four days—that she had been seized with cold chills, after exposure to cold when in a state of perspiration—that violent quinsey succeeded and continued, notwithstanding the application of 50 leeches to the throat and upper part of the breast. More leeches were now proposed, to which she would not, at first, consent, on account of her sense of extreme debility; but ultimately she permitted their application. Fifteen leeches were put to the front of the neck—and a calming potion was ordered. The night was very restless—the sense of suffocation still extreme. 30th. The deglutition a little less difficult—a puriform expectoration was forced out, on expiration, mixed with blood. The respiratory murmur was very faintly heard throughout the whole of the fore part of the chest. All the bad symptoms, described above, continued. She died, suffocated, at 7 o'clock in the morning of the 31st.

*Dissection.* The mucous membrane of the pharynx and larynx presented a vivid redness, and the vessels were finely injected. This redness did not proceed down the œsophagus, but was prolonged in the trachea. On the left side of the larynx there was an ulceration resembling a venereal chancre. The epiglottis was red and thickened, as were the ligaments. The surrounding cellular substance was considerably thickened, infiltrated, and gorged, so as almost to obliterate the rima glottidis. The cavity of the larynx was filled with ropy mucus. The tonsils were ulcerated. All the anterior part of the neck was infiltrated, even the thyroid gland. The lungs were crepitous, except at one part, where their tissue was compact and easily lacerable. The mucous membrane of the bronchiæ was injected, inflamed, and covered with frothy mucus.

*Case* 2. Elen. Lemindre, aged 34 years, had been in the Hospital COCHIN, for disease of the heart, when, on the 23rd February, she was seized with a violent rigor, followed by vomiting. 24th. An erysipelatous eruption on the face—tongue red—thirst urgent—skin hot—pulse frequent. 25th and 26th. The erysipelas extends to the hairy scalp and to the neck—the eyes completely closed. 27th. The progress of the erysipelas continues—complains of acute pain in the throat—difficult deglutition—respiration impeded. The patient refused to have leeches applied. 28th. The swelling and inflammation very considerable on the anterior part of the throat, threatening suffocation. The patient was unable to expectorate, and made constant efforts with her fingers to disentangle the mucus from her throat. 1*st March.* The swelling of the neck enormous—the suffocation imminent. She now consented to the application of leeches; but it was too late. She died in a state of asphyxia two hours after their application.

*Dissection.* The mucous membrane lining the mouth, larynx, and pharynx, red and inflamed. The epiglottis and its ligaments very much thickened—the rima glottidis nearly annihilated by the swelling of the surrounding parts and the tough mucus covering it. The cellular tissue of the larynx, neck, and face, injected, red, and œdematous. The lungs were, generally speaking, sound.

*Case* 3. Charles Gurnier, having had several catarrhal affections, was in the Hospital Cochin for rheumatism, when, on the 10th Nov. 1822, he complained of sore throat, and presented the symptoms of acute angina. On the 11th the fever was high; but the hospital-assistant, who did duty for the physician that day, contented himself with applying mustard pediluvia. 12th. The symptoms were very severe—the breathing difficult—considerable swelling of the tonsils—difficult deglutition. 18 leeches to the throat. 13th. No alleviation of the symptoms—was delirious last night. 14th. He was in a comatose state—lips livid—breathing most laborious—pulse rapid. 15th. Died this day.

*Dissection.* The rima glottidis was greatly diminished, owing to the swelling and infiltration of the surrounding parts. The surface of the tonsils was ulcerated. The mucous membrane of the pharynx was highly injected—the *cellular* membrane surrounding the larynx and pharynx was œdematous, and presented even purulent matter when squeezed. The *mucous* membrane of the larynx was covered with muco-purulent matter—was highly injected, but much less red than that of the trachea and bronchia. The left lung was crepitous—the right was infiltrated. The other morbid appearances were unimportant.

*Remarks.* The term angina *œdematosa* appears to be unnecessary. The œdema clearly constitutes no fundamental character of the disease. It is an effect or consequence of the acute inflammation going on in the parts. It is abundantly evident that, in all human probability, death might have been warded off, in all the above cases, by timely and vigorous antiphlogistic measures. We do not agree with the reporter of these cases, in considering general bleeding as far inferior to local, in laryngeal inflammation. We are quite convinced, from attentive observation, that general bleeding should always accompany the local, and it should be carried to syncope, in order to check the inflammation as quickly as possible. The other antiphlogistic measures are little attended to on the Continent, and the want of success there is partly owing to such neglect.

We have said nothing of laryngotomy in such cases. We are convinced, however, that many lives might be saved by resorting to the knife when other means have failed. There was no organic mischief, for instance, in the cases above detailed, which was irreparable. A free respiration would have preserved life, and the consequences of inflammation would have disappeared in a few days. Death, in all the three instances, clearly resulted from the mechanical obstruction to the respiration, and not from any irremediable disorganization of vital or other parts. Tracheotomy, it is true, is a formidable remedy;—but "ad extremos morbos extrema remedia exquisitè optima."—*Hippocrates.*

*P. S.*—Since the above was translated, we have perused a well written paper, on laryngitis, by Dr. Beck, of New York, in the 12th Number of his journal, from which we shall make a few extracts. Speaking of general blood-letting, he observes that, practical writers have not attached that importance to the remedy which it justly deserves. He attributes, and very justly, the want of success from this measure, to the manner of employing it. Unless it be carried so far as to make a decided impression on the system, as evinced by syncope, the relief will be inadequate to the exigency. "The very same reasons which induce me to consider that the ordinary abstraction of blood can be attended with no great benefit in this disease, convince me of the high and unequivocal advantages which result from carrying it *ad deliquium.* When this effect is produced, the most distant, as well as the most minute parts of the system, come within the sphere of its influence. It not merely moderates, but suspends and alters existing actions, at the same time that it relaxes spasm—a symptom which, in a greater or less degree, always accompanies inflammation of the larynx." In these statements we entirely agree. As to effusion having taken place in the surrounding cellular texture, we do not think it is any proper objection to blood-letting—*while difficulty of breathing continues.* It is this difficulty which endangers life—it is the want of access of air to the lungs that occasions death. Blood-letting is the surest means of relieving the one and preventing the other.

Dr. Beck relates a few cases of this formidable disease, of which we shall take some slight notice in this place.

*Case* 1. Dr. Hoffman, of the United States' Navy, was suddenly called to a gentleman whom he found in a state of threatened suffocation. The patient's countenance expressed extreme anxiety—the neck was denuded—the larynx moving violently up and down—eyes staring—face flushed and covered with sweat—his whole frame was agitated—the difficulty of breathing insufferable. The only expression he could get out was—"can't breathe—can't breathe." A vein was opened in each arm, and bled freely—but death took place in a few minutes after Dr. H. entered the House.

It was now ascertained that he had only complained of sore throat the day before, but was not prevented from following his usual avocations. Believing that the complaint arose from relaxation of the palate, he had used cayenne pepper for relief a short time before Dr. H. was called in.

*Dissection.* The mucous membrane of the fauces appeared slightly inflamed, while that of the rima glottidis was so much inflamed, injected, and infiltrated with serum, "as effectually to conceal and shut up the passage to the wind-pipe." This condition obtained throughout the larynx, and traces of inflammation extended down the trachea as far as its bifurcation.

Here we think it is evident that bleeding copiously would have saved the patient's life in the first instance; and that tracheotomy would have offered a very considerable chance of success, even at the late period when Dr. Hoffman was called in. There was no irreparable mischief produced by the inflammation. The fatal event was produced simply by the strangulation.

*Case* 2. This was in the person of Dr. Francis, one of the Editors of the journal from which we quote.

This gentleman had been complaining, for three or four days, of soreness about the fauces, with some degree of thirst, accompanied by considerable difficulty of deglutition, but without any cough or expectoration. 17th November, 1823, the patient was seized with an increased sense of tightness, attended with pain, difficulty of breathing and swallowing, and sense of strangulation. Venesection to 40 ounces, after which an emetic of tartarized antimony and ipecacuanha. The emetic did not act. An ounce of sulphate of soda was then administered. 7 *in the Evening*. No abatement of the symptoms—pulse full and strong at 120 —skin hot and dry—dyspnœa still continues. Twenty ounces of blood were abstracted. 18th. *Morning*. The uneasiness this morning is excessive, in consequence of the difficulty of breathing, and the frequent returns of sense of strangulation. Considerable soreness was felt when the larynx was pressed externally. Sixteen ounces of blood were taken from the arm, and solution of tartar emetic was ordered. A blister was applied to the throat. *Evening*. No abatement of the symptoms. Sixteen ounces more of blood were taken. 19th. No amendment. Sixteen ounces of blood from the arm. Leeches to the throat—blisters to the chest—antimonial solution continued. *Evening*. Sixteen ounces of blood abstracted from the arm—ten grains of calomel. 20th. Little or no amendment. Sixteen ounces of blood abstracted. Four grains of calomel and six of James's powder to be given every three hours. 21st. No material alteration. Continue the calomel and James's powder. 22nd. Increase of general excitement. Twelve ounces of blood to be taken. The calomel to be continued. 23rd. Patient feels a slight moisture in the mouth and throat. In the course of the day the gums shewed the influence of mercury. The powders to be discontinued. For three or four days after this the patient was still in a precarious condition, and required repetition of bloodletting, with antimonials and blisters. A copious expectoration ensued, and lasted for two or three weeks, after which it gradually declined, and the patient recovered his usual health, but his voice still continues hoarse.

The patient was attended by Drs. Hosack, Post, M'Lean, and Beck. We acknowledge that there is more difficulty in treating the case of a medical man than of any other. This may account for the deviation from the didactic precepts laid down by Dr. Beck himself. Syncope was never produced but once by the bleeding, and that on the 13th day of the disease. To obtain all the benefits derivable from this measure, syncope should have been induced each time the vein was opened during the first days—or rather day of the disease. In cynanche laryngea we maintain that—

Nothing is done while aught remains to do.

Every time the pulse rises and the sense of suffocation returns, the general bleeding should be carried to syncope, or complete relief of the symptoms, whatever the quantity of blood taken may be. This treatment is less formidable in reality than in appearance. We are thoroughly convinced that, in the case before us, much less blood would have been lost, had it been more copiously abstracted in the beginning.

We shall here introduce, in illustration of our remarks, a few of the particulars of a very interesting case in which we were lately concerned, and which we hope will be published in detail by the intelligent surgeon (Mr. Bullen, of Ipswich) who had the patient under his charge.

A gentleman residing in Ipswich, aged 60 years, had had several attacks of inflammation about the larynx and trachea, which always gave way to mild means, as leeches, blisters, cooling purgatives—and especially to brisk doses of the vinum colchici. Early in the spring of the present year he experienced a rather severe attack, which had nearly subsided in consequence of the usual means, when, from imprudent exposure, the tracheal inflammation was renewed with unusual violence, and this time it resisted the remedies which had hitherto subdued it—namely, leeches, blisters, purgatives, antimonials, and colchicum. The relapse happened on a Wednesday night, and by Saturday morning the gentleman's life was evidently in jeopardy. A despatch was sent off to London, and early on Sunday morning, the writer of this article reached Ipswich. The patient had been propped up in an arm-chair all the preceding night, being quite unable to deviate from the perpendicular posture. The breathing was now very laborious, and accompanied by the rattling in the throat, which is the usual harbinger of death. The cough was incessant—the expectoration consisting entirely of thin bloody mucus. The pulse was 120, small and feeble—the skin cool, and covered with clammy sweat—the face pale, excepting a circumscribed spot on each cheek, which was blue, as were also the lips—the tongue was furred and dry—the thirst urgent—the muscular strength entirely prostrate. No stethoscope was at hand; but the "râle muqueux" could be clearly heard with the naked ear when applied to any part of the chest. The patient was unable, by the utmost effort, to inspire more than a very small quantity of air.

Now, considering that this was the fourth day of a *relapse* of tracheal and bronchial inflammation, in a person of 60 years and upwards, whose constitution had been much cut up by military service in various unhealthy climates ,the symptom sappeared to authorise the supposition, that effusion into the bronchial tubes and the air cells had obtained to an extent that allowed little, if any, hope of recovery. This was the opinion of the reporter and of Mr. Bullen, though it was determined not to give up remedial measures till the last moment. The existing state of the symptoms entirely precluded farther depletion at the moment. With the view, however, of promoting expectoration, and also of exciting some reaction in the skin and vascular system, the liq. ammon. acet. with five grains of carbonate of ammonia, were ordered every two hours. More blisters were applied to the chest. The patient's sufferings gradually increased—there seemed to be a complete collapse of the system—and he appeared to be sinking fast. About three o'clock on Sunday he desired to be propped up in bed, as he felt himself dying. He was put in bed, but his sufferings increased there. There appeared, about this time, a slight increase of fulness in the pulse and of temperature in the skin. It was therefore determined, more on the principle of Euthanasia than with any hope of saving life, to open a vein and try the effect. Mr. Bullen did so. The blood flowed freely, and when about 16 ounces were in the basin, the patient declared that his sufferings were mitigated. The basin was set aside and another substituted. Before the second basin was filled, the blood in the first shewed an intense crust of inflammation. By the time that 32 ounces had flowed, the rattling in the throat disappeared, and the breathing was considerably relieved. When 50 ounces had been abstracted, syncope took place, and lasted some time. When life returned, the cough, dyspnœa, and rattling, were no more. The pulse was now at 86, and soft. All the blood exhibited marks of the most intense inflammation. This calm did not long continue. The symptoms gradually rose again, and again the vein was opened. Nothing short of syncope was effectual—and venesection was again carried to that point, without fear. In 12 or 14 hours from the time that the depletion was commenced, the amount of blood drawn amounted to more than 90 ounces—and the whole of the formidable symptoms were subdued. The expectoration changed from blood to tough clear phlegm—from phlegm to mucus—and, from mucus, to a copious puriform fluid. On Tuesday morning, the reporter left Ipswich, the patient being in a fair way of recovery. Two or three days after this the threatening symptoms again recurred, but suddenly gave way, in consequence of the patient falling into a long fit of syncope on getting out of bed to have a motion—a brisk diarrhœa having taken place. From this time he gradually, but slowly, advanced to convalescence, and, finally, to health. The foregoing is but an outline of a very instructive case, which we hope Mr. Bullen will publish in detail. It proves that, in some instances, at least, general blood-letting is far superior to local, where the respiratory apparatus is concerned. The case shews also, that we may have those symptoms which are usually considered as indicative of fatal effusion in the chest, and yet there may be only inflammation or congestion of the vessels of the mucous surfaces, with such an effusion into the bronchia as is remediable by active depletion followed by expectoration. We hope this case may prove useful to some of our readers, when placed in circumstances similar to the above. There never was a case that excited more lively interest at the time, in the minds of patient, practitioners, and friends. Its termination was as gratifying as it was unexpected.

---

2. *Fatal Irritative Fever.** A woman of strong healthy constitution,

* Mr. Anderson. Med. and Phys. Journal, Feb. 1825.

whilst in the daily habit of washing and dressing a suppurating wound in the neck of one of her children, complained of pain and tenderness in one of the joints of the middle finger, the knuckle of which was observed to be swelled and inflamed. There was also perceived a small scratch or wound on the inside of the finger. The inflammation subsided by suitable applications; but, on the following day, she felt pain about the clavicle of that side, extending to the ear. She had also become feverish. Next day the pain had increased, and, in the evening, she was seized with delirium, which went off in the morning, but returned next night. The following day she was seen by Dr. Babington, and, in the succeeding day, Mr. Travers joined in consultation. She was now delirious, and the pulse very rapid. Mr. Travers made a deep incision into the joint of the finger, suspecting the irritation of matter under the sheath of the tendon. A very small quantity of matter was evacuated, but the symptoms were not relieved by the operation. She was bled, and took purgative medicines. Twenty-four leeches to the hand and arm, with constant fomentations. Next day she was bled again, and anodyne medicines were given, but without any diminution of the delirium or watchfulness. In this manner she continued for some days, and, as she was getting weaker, bark and acids were given. Her restlessness and incessant delirium went on till she died, which event was protracted till the completion of three weeks from the accession of the symptoms. On examining the hand and arm after death, the tendons in the palm of the hand were found in a sloughy state, and a collection of matter had formed under the ligamentum annulare of the wrist; but the whole of the arm appeared free from disease.

It is curious that, in this case, the servant who had fomented and poulticed the patient's hand, was attacked with violent inflammation and swelling of the hand and fingers, afterwards extending to the arm. Several collections of matter were formed and opened. Her health suffered so severely from this attack, as to excite, for some time, considerable apprehensions for her life.

This is not the only example where matter from a living body will act as a poison on the body of another person.

---

3. *Inflammation of the Cellular Substance.** Mrs. Craig, aged 30, was admitted a patient into Queensberry Hospital, on the 2d September, 1821, under supposed fever, and complaining of the usual symptoms, as headache, pain of loins, nausea, &c. Pulse 128, full and bounding—tongue foul—bowels costive—heat much increased,—face flushed—eyes suffused. Was seized three days before, while at work, and ascribed her complaint to cold. She had been bled, and had taken some opening medicine. Eighteen months previously she had been affected with syphilis by a child she suckled, for which she had taken mercury and was supposed to be cured. Yet, about three months before her present

* Dr. Duncan. Med. Chir. Trans. Ed. Vol. 1, p. 451.

illness she was delivered of a still-born infant suspected to have been syphilitic. 3d. Much relief from the abstraction of 20 ounces of blood, (which shewed the inflammatory buff) and from the operation of a cathartic. She now complains of severe pain of the right shoulder stretching down the breast and impeding her breathing. The parts affected are much swelled—pulse 112—inclination to be sick—tongue still loaded—eyes suffused. Although pulmonic inflammation was at first suspected, it was now ascertained that the seat of the local complaint was the right arm and the soft parts on the right side of the chest. Antiphlogistic treatment, local and general, was pursued. 4th. The swelling and tenderness much diminished, but she complains of a sense of tightness across the chest checking respiration—pulse 130 and full—tongue still loaded and tremulous—thirst urgent—bowels very free. 30 ounces of blood from the arm shewed the buff, and much relieved the dyspnœa—a blister. 5th. Pain less—inclination to vomit—bowels free—pulse 116, less full.—Effervescing draughts. 6th. Nausea abated—but there is still pain in the arm, increased by pressure—pulse 100—tongue clean—bowels free. Two ounces of port wine, in the form of negus, to be given from time to time. After getting a little of it she became delirious—the pulse rose to 116, full and strong. The wine was discontinued, as Dr. Duncan had often observed that fever patients who had been treated by free depletion, do not bear stimuli. 7th. Nausea and pain of arm continue—stools frequent and frothy, with tenesmus—pulse 114—great thirst—tongue cleaning. 8th. Pain of side impedes breathing. Venesection to 20 ounces, with considerable relief—blood cupped and buffed. 9th. No complaint except pain of arm and side—stools numerous and fœtid. Bolus of calomel and jalap. On the 10th, 11th, and 12th September, she had little complaint and was considered con alescent; but on the 13th she had a renewal of fever, with more violent local affection, the right arm being much swelled, and hard. A tumour, the size of a pigeon's egg, was observed above the right clavicle, not painful on pressure—pulse 120 and full. Venesection gave relief, and the patient was again considered convalescent. 19th. Another accession of fever—and pain, which was again relieved by venesection. On the 1st October, there was a slight, and on the 6th, a very severe accession—pulse 120 and full. Twelve leeches—fomentations. 8th. It was again deemed necessary to apply twelve leeches, in consequence of the pain of the *left* side. On the 11th the tumour abovementioned had become softer, and now, for the first time, slight cough, with copious easy expectoration was observed.

"I suspect that at this time the purulent matter which had formed in the cellular substance of the parietes of the chest external to the *pleura costalis* had, by adhesion and erosion of that membrane, found its way into some of the bronchia, and that in fact it was this pus which constituted the copious, easy expectoration discharged by slight cough. This idea of a communication between the external abscess and the pulmonary passage was confirmed on the 14th, when it was reported that the tumor on the sternum crepitated. The fluid was now expectorated, and air occupied its place. On the 15th, by the progressive evacuation of pus, air had entered into other tumors of the right side; distinct crepitation was perceptible on handling them, and a gurgling rattling was heard when the patient breathed. On the 16th, it was reported that the cough was very severe, without expectoration; emphysematous tumor on the breast increased in size, and the tumors on the side softer. By the assistance of a stethoscope, the respiration was audible in the left side of the thorax, especially on deep inspiration, and accompanied on coughing by considerable rattle, as of air passing through fluid; pulsation of the heart also audible; and, about the middle of the sternum, there was something like egophony; and on the right side, air was heard rushing into all the tumors, with very distinct pectoriloquism in that on the neck above the clavicle, and in the other upon the sternum. The expectoration was much diminished on the 15th, and altogether suppressed on the 16th and 17th, and the patient was dreadfully oppressed with dyspnœa, when immediately after the visit on the 17th, and before I had left the Hospital, I was suddenly called to her, and found that she had just brought up at once about three pounds of pure thick pus, like thick yellow cream, unmixed with froth or mucus, shewing that it had not been diffused through the cancelli of the lungs, but had made its exit through a bronchial tube as through a fistula." 461.

Before this time, our judicious author "had wished to make an external opening into the abscess," having observed that the patient was very much oppressed whenever the expectoration was less abundant, and dreading that it might sooner or later cause suffocation. A lancet was now plunged into the tumour on the side, but without success. On the 21st, however, Mr. Wishart made an opening, when both pus and air were discharged by the wound. 22d. Slept well—cough continues—and two ounces of pus expectorated this morning. Emphysematous tumour on the sternum much diminished in size, and the contained air can be pressed into the thorax, whence it returns as soon as the pressure is removed. There is also a small emphysematous tumour in the neck. On removing the plug from the wound, ten ounces of pus were discharged—in the evening, eight ounces more were discharged. During the latter days of October, the daily issue of pus varied from 10 to 20 ounces. In November it lessened—and on the 18th it was reduced to four ounces. It ceased altogether after the 20th. On different days, it seemed to come from different cavities, according as their communication with the external orifice was open or obstructed. From this period (20th November) hectic fever was established. She left the hospital, at her own desire, on the 3d of December, and died on the 11th of the same month.

*Dissection.* The body was accurately examined by Dr. Craigie, and a minute account of the dissection is given; but this we cannot introduce here. Suffice it to say that the ribs were carious, and that the cavities of abscesses were found within, communicating with some of the bronchial tubes—hence the expectoration of matter from the lungs, and the passage of air from the bronchia to the external parts of the chest. The nature of the case will be understood from Dr. Duncan's comment at the end.

" This case, which, so far as my reading extends, is without a parallel in the annals of medicine, is in several respects remarkable.

" It illustrated the progress in the formation of circumscribed emphysematous tumors, in which the air was confined in cysts formed by adhesive inflammation, and bearing the same relation to common diffuse emphysema that phlegmon does to diffuse cellular inflammation, of which it will be the object of another communication to give some account.

" The utility of the stethoscope, in facilitating the diagnosis of pathological states of the lungs, was placed in a very strong point of view. For not only the rushing of the air into, and out of, the tumors, was most distinctly heard during expiration and inspiration, but in no instance did I ever observe the singular phenomenon of pectoriloquism more decidedly and unequivocally than in those external tumors, even in that above the clavicle.

" The appearances observed after death confirmed, what is indeed already sufficiently known, the manner in which, by the formation of adhesions, communications between internal organs and the surface of the body take place, without effusion of fluid or the admission of air into the cavities in which they are situated.

" By comparing the history of the case with the appearances discovered in the body, various opinions may be formed as to the cause and progress of the disease. It may be supposed, that the whole originated in syphilis, the poison having affected the ends of the ribs, and rendered them carious, while the pus thereby formed had gradually accumulated beneath the pectoral and cervical muscles, and afterwards penetrated and destroyed the *pleura costalis* and *pulmonalis;* or the same effect may be ascribed to the suppuration of the mamma, with which she had been formerly affected.

" But it appears to me more probable that the disease, on the admission of the patient, was recent and acute, the previous inflammation of the right side acting at the utmost as a predisposing cause. The first series of symptoms indicated diffuse inflammation of the cellular tissue of the shoulder, axilla, neck and side, terminating partially in suppuration, and coincided exactly with those observed in cases to be afterwards related, in which the pathology of the affection was established by dissection. The subsequent symptoms were chiefly caused by the want of an external outlet to the pus after it was formed, which destroyed the intercostal muscles, periosteum of the ribs, and the costal pleura, and at last found its way into a bronchial tube, so that pus formed externally to the ribs was expectorated, and on the contrary inspired air inflated external cysts. This progress of the disease is also confirmed in some measure by cases to be afterwards related." 468.

Dr. Duncan is strongly impressed with the idea that this woman's life might have been saved, if free incisions had been early made in the situation where the ribs afterwards became carious—and if the various tumours had been cut into as soon as they appeared. This supposition seems very probable.

4. *Acute Cephalitis Simulating Hepatitis.** M. H. a Dutchman, 24 years of age, of studious and sedentary habits, had been three years affected with hæmorrhoids, but had been otherwise healthy from youth. For twelve months past, this young Hollander had been deeply in love, but concealed his passion. In the beginning of February, he had an erysipelatous affection of the face, to which he paid little attention, and at this period, the discharge from the piles ceased. On the 17th of February, on returning from the theatre, he experienced a violent rigor, which lasted but a short time. Severe head-ache succeeded, with heat of skin, and copious perspiration. In the middle of the night the patient was seized with acute pain in the right hypochondrium, and difficult respiration. In the morning, our author and another physician found the patient in the following condition :—lies on his back—very violent head-ache—heat moderate, and skin moist—*pulse full and quick*—acute pain in the region of the liver, which was very tender to the touch—tongue covered with a white coat—disgust for every kind of food and drink—had an evacuation in the morning—urine scanty and high-coloured—a slight erysipelatous swelling of the face on the right side. *Bled from the arm to* 12 *ounces—diluents.* At 4 P. M. the hepatic pain ceased—the breathing became free—the pulse weak—but the pain in the head continued—thirst. *Twelve leeches to the anus—pediluvium—emollient glyster.* 19th. At nine this morning, the patient seemed to be doing well—but the cephalalgia continued—he had made no water since the evening before—the erysipelas does not spread. (We do not think our author had much reason for concluding that his patient was doing well at this period.) After a slight slumber, it was found that the patient could not open his right eye, in consequence of the swelling of the eyelids. The urine had flowed, and was limpid—the pulse fell—complains of pain in the right side of the head—tendency to syncope. At seven in the evening he became delirious—restless—and made violent struggles to get out of bed—puts his hands often to his head—answers vaguely to questions—pulse quick and sharp—much perspiration over the whole body. He died at midnight.

*Dissection.* When the cranium was raised, about an ounce of sero-sanguineous fluid escaped—the vessels were gorged with blood—the ventricles were partly filled with reddish serum—the meninges were injected to a vivid redness—and there was a circumscribed spot of inflammation of the brain opposite to the right temporal bone, where a purulent exudation could be scraped off with a scalpel. Every other organ in the body was reported to be sound.

*Reflexions.* "There are many maladies," says "M. Bordot, that defy the resources of our art, and go on to a fatal termination in spite of all our remedies." In this we agree with our author; but we do not think that this can justify us for using inert remedies in a dangerous disease. No man in this country will say that M. Bordot gave his patient any

* M. Bordot. Revue Med. Oct. 1824.

chance in the above case. An apparent inflammation of two important organs in a young man of 24, demanded other measures than 12 ounces of blood from the arm, some leeches to the rectum, and emollient glysters. Such, however, is continental practice—and while it lasts, pathology must indeed flourish!

---

5. *Pulmonic Disease.* Dr. Cobb (Ed. Journal, January) has given a minute, and somewhat tedious description of a state of lung by no means unfamiliar to the eye of him who prosecutes morbid anatomy—namely, hepatization, tuberculation, sero-purulent effusion, and adhesions. On these we need not dwell. The only appearance worth noting was this:—"On following the larger bronchial tube, in the centre of the middle lobe at about two inches from the root, we found, instead of a continuous narrowing, a long cylindrical cavity, two inches in length, in shape and size not unlike the thumb of a glove, with its *palmar extremity* narrowed as well as its *tip*; (why not say *both extremities*?) it was a perfect *cul de sac*, with a lining membrane of a dark reddish blue colour, and which I believe was a superadded substance, and not condensed natural structure of the lung." There were no bronchial tubes opening into the cyst, except the direct and large outlet. There was no appearance of purulent secretion on the internal surface of this cavity—nothing but clear mucus could be squeezed from it.

In the history of the case, which is very imperfect, we see no proof that this young man had ever exhibited the symptoms of phthisis pulmonalis; yet Dr. Cobb assumes it as perfectly incontrovertible that the cavity described above, was that of an abscess, and consequently that this case is an unequivocal proof of the "sanability of consumption." It is hardly necessary to state that Dr. Cobb is entirely mistaken in respect to the cavity described. It was evidently a dilatation of bronchial tube, such as has been repeatedly described by Laennec, Andral, and others, during late years. If the reader will turn to page 453 of the 1st volume of this series (Sept. 1824) he will see a case precisely similar to that of Dr. Cobb's, related by Andral, under the head of dilatation of the bronchia.

---

6. *Hospital Reports.** M. Martinet informs us that 135 patients had been treated at L'Hotel Dieu, in the wards of Recamier, during the last quarter of 1824, of which 106 were acute, and 29 chronic diseases. The deaths 28—one of which took place a few hours after being received into the hospital. The great mildness of the season prevented the development of pulmonary diseases—yet catarrhal fevers were pretty numerous, as were rheumatisms and affections of the head. One case of remittent fever terminated fatally and unexpectedly, in the fourth parox-

---

* Par L. Martinet. L'Hotel Dieu, quatrieme trimestre de 1824—dans les Salles de Clinique de M. Le Professeur Recamier.—Revue Med. Jan. 1825.

ysm, and dissection did not exhibit (according to M. Martinet) any adequate cause for the fatal issue. We shall give some particulars of this case.

Dominic Aubrey, 29 years of age, of lymphatic temperament, having the joints swelled, and the lower extremities mis-shapen, entered the Hotel Dieu, on the 6th November, with all the symptoms of inflammatory anasarca, (leucophlegmatie inflammatoire) and without being able to give any information respecting the history of his complaint. On examination, the body was found to be generally œdematous—and there was pain on pressure with the finger, especially on the thighs and legs. The face was swelled, the eyes dull, with great depression and prostration of strength. The patient seemed to understand questions, but would not answer them except with great slowness. He complained of no local pain; but only of general malaise. The tongue seemed to partake of the general œdema, completely filling the mouth, being white and rather moist. The thirst was moderate—the appetite trifling—the belly enormously swelled, but soft and compressible without pain—bowels constipated—breathing rather embarrassed—cough, without expectoration. The chest examined by aid of the stethoscope offered nothing extraordinary—pulse slightly accelerated—can lie in all positions, but best in supination—has little or no power of locomotion—debility extreme. Says he has been subject to palpitations of the heart—urine clear, but not copious. *Ptisans.* 9th. In the same state. 11th. The same, with a slight diarrhœa which succeeded the constipation. *Ptisans.* 13th. Augmentation of the œdema. To the 18th nothing particular occurred. At this time the breathing became impeded, the diarrhœa increased—an epileptiform paroxysm occurred, and continued half an hour, succeeded by torpor and somnolency. Another paroxysm in the night. 20th. Decubitus dorsalis, and all the symptoms unfavourable. Another epileptiform attack. On the 23d, a paroxysm of the kind described put a period to the patient's existence.

*Dissection.* General œdema. The membranes of the brain perfectly sound, as was the brain itself, and so also the spinal marrow with its coverings. *Chest.* Old adhesions of the pleura—lungs easily lacerable, and greatly infiltrated with serum, being very little crepitous—heart enlarged, particularly its left side—its substance soft and lacerable—pericardium sound. *Abdomen.* Great quantity of clear serum in this quarter, without any albuminous flocculencies. The stomach and intestines, inner and outer tunics, in the most perfect state of integrity, excepting that they were unusually pale in colour. The liver was indurated, but not enlarged.

Among the cases of *apoplexy* in this report, there was one, which shews the uncertainty of our pathology even yet, in this class of diseases. The affection made its debût rather suddenly, in the form of an imperfect sopor, which gave way to stimulants; but, in the course of a few days, fever rose, and the patient, who was 70 years of age, sunk on the 11th day, without evincing any paralysis of the arms or lower extremities, or drawing to one side of the mouth. The only phenomenon in connexion with paralysis was some diminution of sensibility in the members of *both* sides.

On dissection, there was found a clot of blood effused into the right thalamus nervi optici, which had ultimately made its way into the ventricle of that side.

In one case of pleuritic inflammation there was such an effusion into the cavity of the chest as required several operations of paracentesis thoracis for its evacuation. The operation was performed by means of a very fine trocar, which did not permit the introduction of any air into the chest. The patient was greatly relieved by the first evacuation, but a fresh collection of sero-purulent extravasation soon rendered necessary a repetition of the paracentesis. To check the regeneration of the fluid, several emetics were exhibited and blisters applied to the side. By these means the patient experienced a considerable amelioration—the lung became partly permeable to the air, as ascertained by the stethoscope—and that side of the thorax became flattened. In about a month after the second operation, a third became necessary, and was practised. When the report closed, three weeks had elapsed since the third paracentesis, and there was every prospect of an ultimate recovery.

We think it will be granted that had it not been for the certainty with which extravasation was ascertained by the stethoscope, there would have been no paracentesis in this case, and consequently no chance for life given to the patient.

In another case there were phenomena which are deserving of notice. The disease had commenced with slight pain in the side, which was very trifling when he applied at the hospital. On examination with the stethoscope, the following phenomena presented themselves :—the respiration was scarcely perceptible in the left side, which sounded extremely dull—the pulsations of the heart could not be heard in any part of that side, nor under the sternum. They were heard, however, and very plainly, in the right side of the thorax. Nevertheless the pulse was scarcely accelerated—the appetite was good, and the patient only complained of general malaise. Six grains of tartar emetic were exhibited, which vomited him twice, and produced a dozen of motions. A blister was applied to the side. From this period the symptoms of effusion gradually diminished—the sound of respiration returned, as did the cardiac pulsations, and the patient was cured.

The following is a curious case.

*Case.* A young man, 22 years of age, who had come into the hospital for a slight catarrhal fever, was seized, during his stay there with severe dyspnœa of a singular kind. Thus at each inspiration, which was easy enough, the larynx was suddenly depressed—while at each expiration, which was excessively difficult, the larynx was slowly raised. The chest sounded well—the expectoration, which was mucous and opake, was very copious. In spite of leeches, cupping, blisters, opium, antimony, æther, and digitalis, the patient fell a victim to this complaint on the 12th day from its commencement. Dissection gave nosatisfactory elucidation of the living phenomena. The bronchiæ were slightly reddened on the inner surface—the lungs were sound, as were all the other viscera.

Of the cases of chronic enteritis and dysentery, none presented any thing particularly worthy of remark. But the reporter takes this opportunity of promulgating Professor Recamier's opinions respecting the last-mentioned disease.

Dysentery, according to Professor R. acknowledges for its cause, a vitiation of the fluids, which stagnate in the alimentary canal—*i. e.* the bile, the intestinal mucus, and the pancreatic secretion. "Inflammation of the mucous membrane is not primary in this affection; but, secondary, the result of the irritating action of these fluids on the internal membrane of the intestines. The pain is prolonged to the rectum, and excoriations of the anus are sometimes produced, *without any pyrexia being present,* in the same way that we see excoriations of the cheeks and lips in certain cases of ophthalmia and coryza, evidently produced by the acrid secretion from the eyes and nares. If we pay attention to the phenomena of dysentery at the beginning, we shall find the pain first in the colon, and not in the rectum, which it only invades some time afterwards—and finally, that it is to the passage of irritating matters through this last-mentioned portion of gut, that is owing the tenesmus. In fact, when death takes place early and suddenly in dysentery, whether from the disease itself or the supervention of another malady, we find no trace of inflammation in the intestinal canal—but only acrid fluids, which are sometimes so irritating as to cause erysipelas in parts with which they come in contact."—*Revue Med. p.* 23.

The foregoing doctrine of *irritation* in the primæ viæ, being the first link in the phenomena of dysentery, has long been maintained in this Journal, as our readers well know.

*Perforation of the Stomach.* A young lad, 16 years of age, very robust for his years, and often troubled with colicky pains in his bowels, and head-aches for the last six months, had had severe cephalalgia for a fortnight previous to his entrance into hospital, accompanied by vertigo. These increased during the last few days, and his debility was extreme. To these symptoms delirium succeeded, with occasional fits of extreme agitation, especially in the night. Leeches had been twice applied to the epigastrium before he was received.

*December* 29*th.* The symptoms were the following:—*Decubitus dorsalis*—features altered—face pale—cephalalgia, stupid appearance—indistinct utterance—no delirium—respiration slow—dry cough—tongue clean—no pain of abdomen on pressure—constipation—pulse and skin natural. 30*th.* The patient had been greatly agitated in the night; otherwise he is in the same state as yesterday, except that there is more prostration. In the evening, there was loss of consciousness, with profound coma. Slight motion could be excited in the limbs when pinched—the left arm is stiff and extended—general convulsive movements take place occasionally—the jaws are clenched—deglutition impossible—pupils a little dilated—breathing slow and rather sonorous—pulse slow, regular, and full—skin warm and moist. *Sixteen leeches to the nape of the neck and behind the ears—sinapisms to the legs.* 31*st.* Same state, except that the coma is still more profound. The pupils are enormously dilated. Died at 9 o'clock this morning.

*Dissection.* The surface of the hemispheres was remarkable for the dryness of the arachnoid and pia mater, and the flatness of the convolutions. Very little blood in the vessels of the meninges. The substance of the brain was of moderate consistence—a very little clear serum in the lateral ventricles. Nothing else remarkable in the head.

*Thorax.* The lungs were reduced to a very small size, and considerably hepatised—about six ounces of dark-coloured fluid in each side of the chest.

*Abdomen.* Some dark-coloured fluid in the stomach. Its mucous membrane sound, except near the cardiac orifice, where there was an oval aperture the size of a shilling, just where the œsophagus terminates in the stomach. The parts immediately surrounding this aperture appeared perfectly sound. All the other abdominal organs quite natural.

Who will pretend to explain the external phenomena in this case, or attempt to reconcile the symptoms with the *post mortem* appearances? Such instances as these may teach young men caution as well as humility, in their diagnoses and prognoses!

Two cases of hepatitis occurred during this quarter—one of which was soon cured by antiphlogistic measures; but the other proved fatal, notwithstanding the same means were employed. The appearances on dissection, in this case, were as follow:—the liver was greatly enlarged in volume, and descended two inches below the edge of the false ribs. It was covered, on its convex side, with false membranes. Its substance was softer and more lacerable than natural, and it was studded with abscesses, varying in size from that of a pea to that of a nut. These abscesses were not lined by any membrane, and they contained a homogeneous fluid, of purulent consistence and colour. Some portions of the liver presented a marbled appearance. There was bile in the gall-bladder. The symptoms of this case are not stated; but such disease we have seen many instances of in this country.

*Case.* A young married woman had for many years, a tumour situated in the right hypochondrium, extending to the linea alba, and shewing an external protuberance. It was of a round form, hard, immoveable, and not painful on pressure. M. Recamier having ascertained a fluctuation in the tumour, considered it an encysted dropsy of the liver, and determined on puncturing it. For this purpose, a small trochar was pushed into the most depending portion of the tumour, and gave issue to a limpid aqueous fluid. The operation was followed by complete success. All the symptoms occasioned by the presence of the encysted tumour in the abdomen vanished, and the patient was discharged the hospital cured. An analysis of the fluid shewed neither albumen, gelatine, or mucus, but, on the other hand, a matter analogous to osmazome.

One case of small-pox terminated fatally, and, on dissection, the internal surface of the trachea and larynx was found covered with an eruption resembling that on the skin. The lungs were hepatised—the bronchia red.

---

7. *Cystitis Hepatica, fatal.** A man, ætat. 43, was seized with severe pain in the belly, accompanied by pyrexia and restlessness. His wife administered the usual remedy on these occasions—a glass of hot gin grog. The pain increased, and Dr. Scott was sent for. On minute examination, the pain was found to be principally referable to the region of the gall-bladder, or thereabouts. The exact part inflamed was not recognized, but it was naturally inferred that it was a portion of the peritoneal coat of some viscus in the region abovementioned. Blood was therefore detracted, and proved buffy—but no mitigation of pain ensued—except that which was diffused over the abdomen. Purgatives, also, failed for nearly two days to open the bowels, and, at last, only brought away black and watery motions. Meanwhile, the pain continued fixed in the right hypochondrium, in spite of blisters, repetition of the bleeding, enemata, warm-baths, leeches, &c. Vomiting of dark matters came on, and the patient sunk about three days from the commencement of the attack.

*Dissection.* The dissectors were a little astonished to find no trace of inflammation or other disease in the whole tract of intestines. These were then removed from the body, and in doing so, they noticed some adhesions between the mesocolon and apparently the lower and concave edge of the liver. Their attention was, therefore, directed to this point, and the first thing that attracted their notice, was "the prominency and size of the gall-bladder." It felt large, and evidently contained a calculus. The adhesions alluded to, existed between this viscus and the mesocolon, the adjacent parts of the liver being sound. There was no calculus in the ducts, which were healthy. The parietes of the gall-bladder itself were thickened to the extent of half an inch. The stone was about the size of a green olive, surrounded by an inky fluid. It was sparkling and resplendent, when held near a candle—its whole outer edge rough, and the angles of its crystals very sharp, and well-calculated to inflame and irritate the lining membrane of the gall-bladder. No other organ or part was diseased.

Dr. Scott appends some sensible remarks on this case. The altered condition of the bile, as it appeared in the gall-bladder, duodenum, and ejections by the mouth and by the rectum, can only be accounted for by the irritation in the gall-bladder, producing disordered secretion in the liver. The latter organ, indeed, is readily deranged in function by irritation in any part of the digestive apparatus.

The cause of death cannot be looked for in any disorganization presented by dissection. The thickened condition of the cystic coats existed four days before death, without producing any disturbance in the

---

* Dr. David Scott. Ed. Journal, April, 1825.

system. It was, therefore, the *pain* which caused death, by deranging the functions of the vital viscera through the medium of the brain and nervous system. Here, an important practical consideration presents itself. It must be evident that, could the nature of the disease have been foreseen, the patient's life might have been saved, in all probability, by the *free exhibition*† of anodynes. But the exact nature or seat of the malady could not, we will say, be ascertained during life. What then? We reiterate, what we have often said before, that practitioners are too much afraid of opium in inflammations, especially of the abdominal viscera. Yet after copious bleeding there is not the smallest danger in its administration, especially if combined with calomel. If this combination had been given in the case in question, the man's life would have been saved. But practitioners seem, in general, to have but one idea in inflammation—depletion, depletion. All other considerations are absorbed in this. The pain, and its consequences on other organs and systems of the body, go for nothing. Bleeding and purging are the catholicons. Yet such cases as the above are calculated to open men's eyes and awaken reflexion. Dr. Scott himself—rather too late it is true —came to a proper train of reflexion on the catastrophe which passed before his eyes. The irritation and pain, he observes, "had so run down the machinery of life, as to have caused the extinction of it." And again, "When the force of inflammation, therefore, has been greatly broken by blood-letting, then sedatives with purgatives, may be safely tried for the reduction of the rest." In this, we agree with Dr. Scott, and regret that he did not pursue this plan earlier in the disease, and with a more liberal hand. The *quantity* of opium should never be considered in such cases, but only the *effect*. It must be given so as to subdue pain and irritation, whatever may be the magnitude of the dose. It is, indeed, astonishing, how much opium will be borne in painful affections, without producing any disorder of the head or nervous system. The narcotic seems to be expended on the pain, as it were, and to leave all other feelings and functions unmolested.

---

8. *Seat and Cause of Epilepsy.** A child, 14 months old, and not yet weaned, was seized with convulsions, violent and general, at 5 o'clock in the evening of the 16th December, and without premonitory symptoms. M. Menard being summoned, recognized an attack of epilepsy, as characterised by loss of sense, convulsions, foaming at the mouth, clenching of the fists, &c. An emetic was administered to unload the stomach. The child vomited—the convulsions ceased, but were succeeded by comatose sleep and stertorous breathing. The night and succeeding day (17th) passed " without accident." The child occasionally took the breast, but could not sit up—the eyes were dull—there were some tremulous motions of the lower jaw—pulse slightly

† Opium and camphor were ordered, and appeared to do good, 16 or 18 hours before death; but they were too late.

* M. Alphonse Menard, Revue Medicale, Mars, 1825.

febrile. 18th. Grinding of the teeth last night—pulse tense and vibrating this morning—takes the breast—violent pulsation at the anterior fontanelle. Our author properly recommended leeches and cold applications to the head—with purgatives internally; but was frustrated by the idle apprehensions and prejudices of the parents.* Only a dose of castor oil could be given. In the evening, the cerebral excitement appearing to make progress, leave was given to apply six leeches behind the ears—and a bladder of cold water to the head. 19th. The leech-bites bled all night—the child slept profoundly. This morning he passed a living lumbricus, and a clamour was instantly raised against the physician, for not foreseeing the birth of the stranger! He was accused of directing all his attention to the poor child's head, while the enemy was in the abdomen! Nevertheless the cerebral phlegmasia continued its course. The pupils became dilated and inobedient to light—neither the intestines nor skin could be excited by purgatives or blisters. 20th. The convulsions returned with great violence—the bladder was paralysed—priapism—the action of the heart was tumultuous—the pulse at the wrist imperceptible—death at 11 o'clock at night.

*Dissection.* The spinal marrow was opened from the occipital foramen to the sacrum. There were red patches here and there on the dura matral covering—increasing in intensity as the examination was carried upwards towards the head. At the basis cranii, and for a space of four inches downwards, there was a small quantity of pus between the arachnoid and dura mater. The spinal marrow was much softened, and, in some places, quite diffluent. The arachnoid was healthy. In the head, the dura mater was strongly adherent in several places, particularly about the occipital region. The cerebellum was softened and diffluent at its base. There was no trace of inflammation in the cerebrum. The dura mater was thickened and much injected—and between it and the arachnoid, over the hemispheres, was a thin layer of greenish purulent matter. The arachnoid itself was sound. The abdominal viscera were all healthy.

*Reflexions.* Our author is of opinion, from this and many other cases which have fallen under his notice, that the cerebellum is the seat of epilepsy, and that the affections of the spinal marrow or coverings are consequent on the cerebellic inflammation. In these cases, there are almost invariably the following phenomena, viz. retraction of the head backwards—retention, more or less complete, of the urine, and priapism. He could relate more than 20 cases of this kind. It is to be observed, however, that these observations apply only to acute epilepsy. It can-

* We often hear of these contraventions, and cannot but think that they generally result from the *manner* of recommending the measures on the part of the medical attendant. There should be no appearance of hesitation or doubt. After due deliberation, the means of relief should be placed before the view of the parents, and if any reluctance be manifested, it should be mildly but firmly hinted, that the responsibility then rested on their shoulders. This hint generally brings them to their senses.

not be supposed that, in those temporary and periodical seizures of epilepsy continuing for years, there should be inflammation of the cerebellum. But there may be some other affection—irritation, for instance, in that portion of brain.

---

9. *Concussion of the Spine.** This case is related by Mr. Dundas, with the view of illustrating the doctrines advanced by Bell, Spurzheim, and Magendie, respecting the separate offices of the anterior and posterior roots of the spinal nerves:—the one being now pretty clearly ascertained to be for motion, and the other for sensibility.

*Case.* Francisco Cæsario, 35 years of age, fell on his back from a scaffolding 20 feet high. He was stunned for awhile by the fall; but, on recovering, found his left side, from the shoulder downwards, deprived of motion, sensation being unimpaired. On the other side of the body the case was reversed—the power of motion was complete, while sensation was extinct. This loss of sensation, on the right side, was so complete, that, at the end of three months from the accident, a lancet might be pushed into the muscles without occasioning any pain. Yet, over this side, the power of voluntary motion was quite perfect. The muscles of the left, or paralysed side, were flaccid and wasted. The temperature of the *senseless* side was some degrees lower than that of the *paralytic*, which was morbidly sensible. From the fourth cervical vertebra upwards, motion and sensation were perfect on both sides—and the line of demarcation was so exactly drawn, that it might be defined by a pack-thread surrounding the neck. The energies of the patient's mind were not impaired. His bowels would not act without purgatives. The motions were unnatural, varying in appearance from light clay to pitchy black. No indication of violence could be detected in the vertebral column, except a slight degree of tenderness, on pressure, over the tenth dorsal vertebra, unaccompanied by any perceptible swelling. *Active purgatives every second night—stimulating enemata—blisters to the spine.* The nux vomica was next administered; and when the dose amounted to 20 grains, the patient complained of spasmodic twitches of the muscles of the *right* side, and of the face and throat, whilst the *left* side was affected with constant tingling pains, and a most disagreeable sensation of heat throughout the side and limbs. The dose was still farther increased, and the spasms became more violent, and spread occasionally to the left, as well as the right side. Trismus even was induced, the dose of the medicine now amounting to 40 grains. The remedy was discontinued, no good having been produced. At this time a lancet might be pushed into the flesh without occasioning any particular pain.

It is curious that, in this case, the diagonally opposite columns of the spinal marrow must have received injury:—that is, the *anterior* co-

---

* Mr. Dundas. Ed. Journal, April, 1825.

lumn on one side, and the *posterior* column on the other, must be the seat of lesion.

While on this subject, we may mention that Dr. Spurzheim long ago drew the attention of physiologists to the different origins of the spinal nerves, and considered it likely that one was for motion and the other for sensation. In a late lecture on, and dissection of, the brain, by this distinguished phrenologist, Dr. Spurzheim expressed his apprehension that there was yet some fallacy in the experiments of Bell and Magendie, relative to the physiological point in question. He remarks that it is a general law in the animal economy, that wherever an organ is large and the function strong or important, there will be large nerves going to the part, and vice versâ. Now, he says that the anterior roots of the spinal nerves, coming from the cerebrum and destined for voluntary motion, are not much more than half the size of the posterior roots, coming from the cerebellum and destined for sensation. He thinks that, according to analogy with other nerves and functions, the nerves supplying the muscles, where such great power is wanted, ought to be the strongest. For our own parts, we confess that these arguments of Dr. Spurzheim appear to us very weak. In the first place, reasoning must give way to the force of fact and direct experiment. In the second place, we see that the rule which Dr. Spurzheim lays down does not always hold good. Thus the portio dura going to the face, and, which is now unequivocally proved to be a nerve of motion, is very small; while the branches of the fifth pair, destined for sensation about the face, are comparatively very large. These considerations, coupled with the evidence of experiments made on animals, will leave little doubt, we think, that the anterior roots, though small, are destined for motion, and that the posterior, though large, are appropriated to sensation.

---

10. *Hæmorrhagic and Cerebriform Tumours.* A paper was read some months ago, at the Royal Academy of Medicine, by M. Velpeau, on cancerous affections, with the view of shewing that, in these diseases, there are certain morbid alterations in the fluids, to which the attention of the profession ought to be directed. M. Velpeau avers, and probably with justice, that Hippocrates was as well acquainted with the nature of carcinomatous maladies, as the physicians of the present day. This observation, indeed, may be made without paying any great compliment, after all, to the father of physic. We shall proceed, however, to the facts of the paper.

*Case* 1. Rainé, aged 30 years, perceived, on the 19th July, 1823, after a violent corporeal exertion in pushing forward a voiture, a small tumour in his left arm-pit. This tumour remained stationary till the middle of November, when it suddenly enlarged one night and became painful. The pain ceased as suddenly again, and did not return till the month of January, 1824, when lancinating pains were felt in the armpit, shoulder, and arm. These gradually increased, and compelled the patient to apply to several medical men, who could not ascertain the nature of the complaint. Not being able to follow his occupation as a coachman, he presented himself to the Hospital of the faculty on the 12th January. He appeared to be in good health otherwise, and to have a sound constitution. A tumour existed in the arm-pit, the size of a child's head, pushing *forward* the edge of the pectoral muscle, *backward* the scapula, and *upwards* the acromion scapulæ. The colour of the integument was not changed—the arm was swelled and œdematous. Some of the medical officers of the establishment considered this tumour as simply phlegmonous—others that it was aneurismal. Laennec, Boyer, Richerand, Dupuytren, Beclard, Breschet, and other lions of Paris, gave almost as many opinions as there were persons. By the 22nd, the pains had increased to such a degree as to deprive the patient of repose, and oblige him to take opium. The symptoms made progress through the month of March, the tumour becoming larger every day. On the 6th April it burst, and discharged, at one point, some drops of blood. In the night, more blood was discharged. On the 9th, about half a pint was discharged from a new opening. 10th. 12 ounces—11th. A pint of blood.—12th. 28 ounces of blood escaped.—14th. The same quantity. More or less was discharged till the 20th, when the patient was reduced to a state of pallor and yellowness that indicated great danger. The blood always flowed in jets—coagulated in the vessels—and often shewed the inflammatory buff. At each hæmorrhage the patient was relieved in his feelings. These discharges were succeeded by a kind of *molimen hæmorrhagicum*—that is, a sense of heat, thirst, cephalalgia, and general malaise.

A diarrhœa, of a very debilitating kind, now set in, and continued to harrass the patient till the 6th of May, when death closed the scene.

*Dissection.* The arm was infiltrated. The tumour was nearly the size of an adult's head. The ulcerations on its surface had a black and fungous appearance. Its structure exhibited the fibrous and medullary matters found in cancerous tumours. All the structures in the neighbourhood were amalgamated in the diseased mass, except the nerves and blood-vessels. The pancreas was cancerous in part of its structure, as was also one of the kidneys.

*Case* 2. Lucas, aged 42 years, much given to spirituous potations, and of irascible disposition, received, in the month of December, 1823, a blow on the scrotum, which gave him little pain at the time. A few days afterwards he perceived the right testicle enlarged and rather harder than natural, with some shooting pains in the direction of the spermatic cord, which gradually increased at first, and afterwards rapidly. He was put on a mercurial course by M. Dupuytren. While in the Hotel Dieu the tunica vaginalis was punctured, but only some reddish serum issued forth. He was a little relieved for a time, but, in a few days, he became affected with dry cough, for which he left the hospital. The testicle was now the size of a child's head, very hard, and the seat of lancinating pains. The cord appeared sound at its entrance into the abdomen; but some hardness was felt beyond the ring, in the iliac fossa. The patient became afflicted with colicky pains—the cough persisted—the skin assumed a pallid yellow tint. In the beginning of August, 1824, the right leg began to œdematise, and soon afterwards the left, as well as the integuments of the abdomen. These symptoms increased—the respiration became more and more difficult, till, at length, it could only be performed in the perpendicular position—the digestion was lost—in fact, all the functions, except the intellectual, became impaired, and at length annihilated. The patient died on the 29th September, 1824.

*Dissection.* The brain was sound, as was the heart. The lungs were filled with cancerous concretions (according to our author) of all sizes, from that of a large goose egg to that of a small nut. "Il semble que ce soient autant de pommes de terre éparpillées dans le tissu de l'organe respiratoire." They were nearly one hundred in number—appeared not connected intimately with any of the other structures—the intermediate pulmonary tissue was sound—the substance of these bodies was cerebriform, and of various degrees of consistence. The testicular tumour was of the same nature, but rather more complex—and a chain of similar tumour was seen ascending from the abdominal ring up to the diaphragm, communicating a degeneration of structure similar to its own to those parts in immediate contact with the said chain.

*Remarks.* The tumour causing the death of the first patient was evidently a fungus hæmatodes. In the second case, the general disposition to the disease called medullary sarcoma is very remarkable. But we do not see how these tumours can be explained, as to their nature, on the principle of an alteration in the fluids. The fact is, we are ignorant of their cause and nature.

---

11. *Goitre, or Bronchocele.* It will be remembered that we have, in several parts of this Journal, hazarded the opinion, that the cause of bronchocele is some impregnation (hitherto unascertained) in the water used for drink, where the disease prevails. The following very curious history will greatly strengthen this opinion. It is contained in a letter from Dr. Alex. Coventry, President of the Medical Society of the State of New York, to the Editors of the New York Medical and Physical Journal, for June, 1824, No. 10.

the habit that writers are in of copying each other, no such place being known for nearly thirty years past. The village of Utica now occupies the site of the former fort, and if there is a single case of goitre among a population of four thousand souls, it is unknown to me. Dr. Barton, formerly of Philadelphia, in his memoir, and, I believe, President Dwight, late of New-Haven, speak of a family near old Fort Schuyler as instances of goitre. It is now above twenty years since this family removed from land which I now occupy; and, to prevent repetition, I will attempt a short topographical description of the vicinity near Utica.

"The Mohawk river occupies the bottom of a valley, which, from the summit of one ridge to the other, may be seven or eight miles in extent. Utica is in lat. 43° 06′, lon. 75° 13′, about ninety-six miles west from Albany. The bottom of the valley is perhaps 370 feet above tide water, and neither ridge exceeds seven hundred in elevation above the river. On the south ridge, which I suspect is the most elevated, are found quarries of various species of rock from which stones for building are supplied. The beds of the small streams which flow into the south side of the Mohawk, are composed of hard gravel, excellent for repairing roads, &c. On the north side of the Mohawk river, immediately opposite Utica, a flat of clayey alluvion, sufficiently tenacious for the formation of brick, extends nearly a mile, and is joined at its northern edge by a track of gravelly or sandy loam. After an ascent of 25 or 30 feet this last mentioned tract spreads with nearly a level surface for about three quarters of a mile northerly, where it joins the foot of the ridge. This seems to join the hill, and the alluvion may be called a secondary intervale; from the north edge of this, the ridge rises with a gradual ascent for about two miles, and to an elevation of about 600 feet. At the foot of the ridge, wherever a small stream flows, the schist is seen in horizontal layers of a soft friable texture, and, when exposed to the air, slacks into shelly matter, and soon becomes a black crust. This rock approaches the surface on the south side of the ridge, and on the top is found within a few feet, forming the bottoms of cellars, and obstructing the digging of wells. In fact, the body of the ridge on the north side of the valley at this place is a schistus or slate rock, and differs from the south ridge in having no other rock superincumbent. All the streams entering the Mohawk on the north side, at this place, run on a bed of shelly slate gravel, which is useless for roads, as it crumbles into an impalpable black dust. In a south-east direction from Utica, about seven or eight miles distant, and behind the north ridge (as seen from the valley) a small stream arises, which, after running several miles with a west course, turns southerly, cuts through the ridge, and, winding along the low lands, enters the river a few rods below the bridge at Utica. Although, in the summer months, all the water of this stream would frequently run through a tube of four inches diameter, yet it has cut down the north ridge to its centre, having removed an immense quantity of the schist, forming a vast hollow, which, in the vicinity, is called *par excellence* the great gulf. At places, the level flat surface of the rock is exposed for one hundred feet in perpendicular height; the stream of course runs on a bottom of slate rock till it reaches the secondary intervale, from which part to its mouth the bed consists of the slaty gravel before mentioned. On the bank of this stream, where the secondary intervale joins the clayey alluvion, stood the house of Mr. Regel, and the brook still goes by the name of Regel's creek.

"In the spring of 1798 I settled near Mr. Regel, on the west side of the stream, and within thirty or forty rods of his house, but on the secondary intervale, and considerably higher than his location. Regel's family consisted of himself, wife, four sons, and one daughter, all of adult age, and with one exception, troubled with bronchocele. The old man had an enormous goitre reaching from ear to ear, and hanging down on his breast. His respiration was considerably impeded so that I have heard his breathing at fifteen or twenty rods distance. He resided in this place previous to the revolutionary war, during which he was carried off by the Indians: his goitre was then about the size of a doubled fist, but disappeared entirely while he was a captive in Canada, where he was detained two years. On his return the swelling appeared again, and had gradually attained its greatest size. This man had no other ailment and seemed about sixty years of age. His wife had also a large goitre about half the size of her husband's: three sons had moderately sized swelled necks, the daughter rather larger: the latter had passed a fortnight at Saratoga springs, during which her goitre had by measurement decreased one half. The fourth son, named Frederick, had no signs of goitre. I cannot now ascertain whether he had been brought up at his father's house or not: when I became acquainted with him he resided at a distance of three quarters of a mile, at the foot of a hill, by a pure and beautiful spring, the water of which he used.

"There being no well, the rest of the family used the water of the creek spoken of, for all culinary and other purposes. Prepossessed with an idea of the hereditary nature of the disease, I also used the brook water, when towards the approach of the winter, to my no small mortification and vexation, I perceived a considerable goitrous enlargement in the neck of my wife, and an evident thickening in the necks of my two daughters. Then I first began to suspect the water of Regel's creek. The next summer I sunk a well about thirty feet through a gravelly sand, at the bottom of which a quicksand obliged me to sink a curb. The well-water is very fine; and since we commenced the use of it none of my family has been subject to goitre. Regel's family removed to the county of Onandago, and I have been informed recovered from the goitrous swellings: even the old man is said to have got rid of his before death, but this I can scarcely credit.

"A few years after this I leased a small house standing near the brook, to a Mr. Walworth, who had a son about twelve years old. This lad in the course of a few months exhibited appearance of bronchocele: he was sent from home, and his neck returned to its natural size; but in the succeeding season began to enlarge, and the family moved away.

"About the autumn of 1802 I put a small flock of sheep into a pasture through which the stream ran: next spring one lamb proved goitrous: the succeeding season every lamb had a swelled neck, and seven out of eight died. On opening the gland, nothing was perceivable but an enlargement. My next neighbour had sheep in an adjoining pasture, which was watered by a spring: his sheep had no distemper. My flock has been kept where they do not get to the creek water, and are now free from the disease.

"With these facts before me it was not unreasonable to suspect that the water of the stream had a tendency to affect the thyroid gland. I have to regret that no analysis has yet been made of it, but hope in the course of the summer this defect may be supplied: meantime I am tempted to hazard a conjecture as to the material with which it may be impregnated. We know that the schist is not soluble in water, but readily acted upon by the air, with the oxygen of which it seems to have great affinity. This is observable in a dry season, when the surface of the schist will be covered with a white florescence, which on examination is found to be alum. Near Glasgow, in Scotland, a very extensive alum work is carried on, the material for which is found in the schist rock which forms the side, bottom, and roof of an exhausted coal pit. In this situation the slate exposed to a current of air, but protected from the rain, combines with the oxygen, and in process of time forms sulphate of alumine, from which, I believe, the sulphuric acid is procured.

"The crystallization formed on the schist rock, is washed down into the stream by the first rain, and must impregnate the stream with alum: but whether or not this solution would affect the gland, or in what manner, we are not able to say." 166.

We hope these facts will lead gentlemen, resident in goitrous countries and places, to analyse the waters, and endeavour to ascertain the cause of this curious and unseemly disease.

---

12. *Insanity**. The stomach will often be disordered in its function, without any disease of its structure. So will the brain. Wine taken into the stomach will make the wisest man insane for the time; and yet the organ of thought will not be organically affected. On the other hand, the brain may be greatly changed in the structure, and even a portion of it destroyed, without any apparent defect of intellectual function. With this knowledge before our eyes, we can expect but little light on the nature of insanity, from *post mortem* examinations. The fifty dissections performed by Dr. Neumann tend rather to perplex than elucidate the pathological condition of insanity. The morbid appearances were extremely diversified, and in several instances, there were no morbid parts in the cranial cavity, to account for the mental aberration, of which the first case detailed is an example.

* M. Neumann, of Berlin. Account of fifty dissections, &c.

*Case* 1. A watchmaker, 35 years of age, had been afflicted with violent mania for three months before entering the hospital. The cause could not be traced. The patient was attacked with tubercular consumption while under mental derangement, and died two months after being received into hospital. The skull presented nothing particular. The coverings of the brain were healthy, as was the structure of the brain itself. In the fourth ventricle was some clear fluid, as there was also in the spinal cavity. Excepting the lungs, all the other viscera were sound.

*Case* 2. This was an old Hungarian soldier who had been in the Lunatic Asylum for twenty-one years. He died at the age of 60, and was examined. The cranium was every where much thickened, and in the posterior part was upwards of an inch thick. In the course of the sagittal suture, at the side of the falx, between the arachnoid and dura mater, there was a deposition of some cretaceous matters. The cerebral vessels were empty, and there was a considerable quantity of serous fluid in the ventricles. The encephalic mass was firmer than usual.

Here follows another, and a rather different example.

*Case* 3. A postillion, aged 32, became maniacal, without any assignable cause. By proper means this subsided, and then he fell into an idiotic state, in which he continued two years. He died of a colliquative diarrhœa. On examination, the skull was observed to be thicker than natural. The dura mater was easily detached from the bones. On puncturing it a considerable quantity of serum escaped. There was no trace of the arachnoid membrane. A whitish gelatinous mass occupied the space between the dura mater and encephalon, the convolutions of which were compressed. All the ventricles were gorged with serum—the encephalon was a great deal softer than natural. There was nothing unusual observed in the thoracic and abdominal organs.

We think it needless to multiply examples of the various appearances, *post mortem*, in insanity. Many of them were probably accidental, and still more of them consequential rather than etiological. We repeat again that the functions of the brain may, like those of any other organ, be disturbed, without its being possible to discover any alteration in the encephalon or its coverings. It is not, therefore, to morbid anatomy that we can very confidently look for the *causes* of insanity. These are often moral or external; and their first impressions elude the scalpel of the anatomist.

The view taken by Dr. Spurzheim of insanity, is very ingenious, and accords with the phenomena and post mortem researches better than any other doctrine. If it be true, and we believe it is true, that the brain is a congeries of organs or parts, each of which has its separate function in the manifestation of the mental faculties, as well as in forming the organs of sense, then we may easily conceive that one organ or part becoming more developed, and its function more exercised, than is salutary for the general harmony and balance of the whole of the other organs and their functions, aberration may take place. We see this every day. In a man, for instance, who has a strong propensity of any kind—say, by way of example, for religious contemplations, and where this one propensity is allowed to run riot and absorb or overshadow all the other propensities of our nature, the harmonious balance of the various organs and functions of the brain will be disturbed, and mental derangement will be the result. In such cases, we cannot expect to detect the cerebral lesion (if indeed there be any) by the scalpel. Phrenology bids fairest to ascertain the nature of insanity (where it depends, as it very generally does) on moral *causes*, by comparing the faculty most disordered with the organ by which that faculty is supposed to be manifested. Thus, there are many instances of insanity from the immoderate love of offspring among females — pride among men, and so on. Now, if it be proved, in such cases, that the organs or parts of the brain, whose office it is to exhibit the manifestation of these original or fundamental powers or propensities of our nature, should be more developed than in proportion to other parts of the brain, we shall have, at least, some clue to the *predisposition* towards insanity. And as we know that all organs of the body (the muscles for instance) are strengthened by proper exercise of their functions, so, in the mental powers and propensities, we shall often be able to check the domineering influence of *one*, by calling into action the countervailing influence of *another*. These observations appertain only to insanity resulting from moral or mental causes. Corporeal disease, and material lesion of the brain also produce insanity—as where injuries are received, or where intoxication is persevered in—here our post mortem researches are more likely to elucidate the pathology of the complaint.

13. *Phlegmatia Dolens.** The pathology of this disease has caused considerable discussion in this country, as our readers know. Some of Dr. Velpeau's ideas have been anticipated here by our countryman, Dr. Davis—but of this M. Velpeau is ignorant. The French know exceedingly little of what is passing in England—nor do they wish to know. There are a few exceptions, such as M. Breschet and some other able and intelligent men of the French metropolis; but the proportion is very small. Phlegmatia dolens is so very rarely fatal that the morbid anatomy of the disease is very defective. Whether the three following cases, with their dissections, will throw an increase of light on the nature or cause of the affection, we shall enquire farther on.

*Case* 1. Valette, 18 years of age, entered the hospital Sainte Come, on the 19th October, 1823, for the purpose of accouchment. The process of parturition was tedious and painful, but not attended with any

---

* Recherches et Observations sur la Phlegmatia Alba Dolens. Par M. Velpeau, Chef de Clinique à la Faculté de Medecine de Paris. Archives, Octobre, 1824.

circumstance worthy of record. On the third day, when the milk fever was forming, she received some melancholy tidings, which made a strong impression, and the fever was increased. The mammæ were much gorged. On the 5th day, the lochia ceased, and this cessation was succeeded by cough and pain in the chest. This state continued till the 11th day, when the febrile symptoms ceased, and some appetite returned. On the 12th day, it was ascertained that the patient had got some wine the evening before. In the evening a rigor, followed by fever and profuse perspiration. The abdomen was not painful. The appetite was gone—the cough still continued. Each day the above-mentioned phenomena were renewed, with the usual symptoms of an autumnal intermittent fever, until the 16th day, when the fever ceased. *17th day.* The pulse is small—patient feels feeble—tongue dry. On the 20th, the cold chills and fever returned, with pains in the groins, hypochondria, and left side of the pelvis. The abdomen became suddenly distended—the patient was inclined to somnolency. 25 leeches to the abdomen. 21st day, the abdominal swelling is less, but the part is tender on pressure, especially in the hypogastric and inguinal region. No particular alteration till the 30th day, when the appearance of the patient indicated some great internal lesion, the nature of which could not be ascertained. By the 40th day, the cough had ceased. On the 41st day, the left lower extremity was found to be swelled, accompanied by violent pain in the hip and groin, extending ultimately to the whole limb. *43d day.* The whole extremity œdematous. Pressure gave pain only in the groin. Irregular fever took place from time to time—an eschar formed over the sacrum—the strength diminished. On the 59th day, delirium supervened—and on the 60th, death closed the scene.

*Dissection.* There was nothing remarkable in the appearance of the brain, heart, lungs, or viscera of the abdomen. The uterus appeared sound externally, as also the ovaries and fallopian tubes. When the left extremity was cut into, it was found much infiltrated in the cellular tissue. The lymphatic glands of the groin were much swelled and red—the muscles small and pale—the vena saphena sound—the crural vein, attentively examined, was found to be red externally, and its cellular coat thickened. This appearance obtained in all its branches deep-seated in the thigh, leg, and in the hypogastric vein. When laid open, these vessels were found filled with purulent matter, concrete, and adherent to their internal surfaces, particularly in the groin and internal iliac fossa. When scraped off the internal surface of the vessels, the latter appeared sound, being pale and not thickened. The primitive iliac vein was also filled with purulent matter of a grumous and membraniform appearance. This vessel was strongly adherent to the sacro-iliac symphysis by means of apparently inflamed cellular tissue. Pus was found in the inferior cava, from its commencement to its termination in the auricle, which cavity also was full of the same. The ventricle, the pulmonary artery even, presented the same contents, mixed with blood evidently altered in its composition. The tunics of the vessels presented no deviation from health. The same might be said of the arterial system. A few small abscesses were found between the muscles of the leg. The inter-pubian cartilage was softened—and matter similar to that in the veins was found there. The same appearances were observable in the left sacro-iliac symphysis. The coxo-femoral articulation presented some purulent matter of very fœtid quality. There was no disease in the other lower extremity, or its corresponding vessels.

*Remarks.* We really can see no evidence in this case of inflammation of the internal surfaces of the veins. They contained a purulent looking matter, it is true; but when scraped with a scalpel, their internal tunics were healthy and even pale. The fluid found in the veins might have been taken up from parts where a suppurative process was going on, as in the symphyses and sacro-iliac fossæ, as above-mentioned; or it might have been an altered condition of the blood itself. " Cette mattiere" says the author, " est melée à une grande quantité *de sang qui est evidemment alterée dans sa composition.* Ce fluide en effet, offre ici un aspect fort remarquable: il semble etre formé par une grande quantité de petites graines peu consistantes, de couleur variée, qui auraient été melangées dans un liquide noiratre, &c. *Les vaisseaux eux-memes n'offraient aucune alteration appreciable de leurs tissus.*" Whatever then was the nature or composition of this fluid, there is nothing to lead us to the conclusion that it was the result of any affection of the containing vessels.

*Case* 2. Damiens, a woman 35 years of age, of athletic form and stature, entered the Hôpital de la Faculte, on the 10th February, 1824, for accouchment. Two months previously, she had had a fall on her back, which confined her to bed for a few days. Her delivery was quick and easy. During the first three days nothing remarkable occurred. On the *4th day*, fever arose, with deep-seated pain in the pelvis towards the sacrum. *Venesection. 5th day.* She was better—no pain in the abdomen. *6th day.* The pains in the pelvis have returned with greater violence than ever, and seem to radiate towards the loins. *Venesection repeated. 7th day.* The pains are mitigated; but in the evening there was a rigor followed by fever. *Venesection a third time. 8th day.* Better. *9th day.* Ill again. The pulse is hard and quick—tongue white and loaded—feels a dull weight in the loins and in the pelvis. *10th and 11th days.* Same state. Had occasional shiverings. *12th day.* The pains are increased, and shoot down the thighs. The lochia still continues. 30 *leeches to the anus. 13th day.* The lower extremities are much swelled and painful, especially the *left. 15th day.* The breathing is affected—passage of the urine difficult. *19th day.* A diarrhœa has supervened, and the limbs appear much swelled. *20th day.* The lower extremities are again more swelled and *red.* (enflées et rouges*) The

* A curious instance of " Phlegmatia *Alba* Dolens," as the author calls the disease !

belly swelled and painful—evacuations involuntary. She lingered out till the *26th day*, when she expired.

*Dissection.* Head not examined. All the viscera of the thorax sound. *Abdomen.* About two pints of yellowish serum in this cavity. Some red patches on the peritoneum, which was generally thickened, softened, and in some places infiltrated. The uterus was larger than natural, and had some purulent depôts on its internal surface. *One* of the ovaries and corresponding tube were inflamed and even suppurated. There was a puriform fluid found in most of the veins of the left side, hypogastric and femoral. The left lower extremity was extremely swelled and œdematous, with vast abscesses between the muscles. The inguinal glands were double or triple their natural size. Several of the lymphatic vessels contained purulent matter, the same as the veins, as did also the thoracic duct itself. The symphyses (sacro-iliac and pubic) were in a state nearly similar to that described in the preceding case. Nothing is said of the condition of the vessels themselves. We must, therefore, conclude that, as in the former patient, they exhibited no morbid phenomena.

*Remarks.* Here again, we have no evidence of inflammation of the veins.

*Case* 3. Lefevre, 24 years of age, was received at La Maternité for confinement of her second child, which took place eight days afterwards —28th April, 1824. The process of parturition was tedious and painful. Nevertheless the first three days passed without any thing particular occurring. On the 4th day, there were deep-seated pains in the groins and pelvis extending to the abdomen. 40 leeches were applied to the anus. On the *5th day*, finding herself much better, the patient requested her dismissal from the hospital, which was granted. She had scarcely reached home when she was seized with rigors followed by sharp fever. Pains all over the body, but more acute in the pelvis and lower extremities. *6th day.* She was brought to the Hôpital de la Faculté, where she presented the following phenomena:—countenance cheerful—tongue white—thirst—breathing short, but easy—pulse small, quick, and feeble—skin dry and burning—abdomen much swelled with flatus, but not at all painful on pressure—pains in the upper and lower extremities—the latter beginning to shew œdema. *Venesection. 7th day.* Better in the morning; but increase of the pains in the evening. Diarrhœa. *Venesection repeated*—40 leeches to the anus. Delirious all night—diarrhœa very troublesome. *8th day.* All the symptoms exasperated. Breathing short and difficult—epigastrium painful on pressure —inclination to vomit. 30 leeches to the epigastrium. Died at 12 o'clock.

*Dissection.* Cranial and thoracic viscera all sound. Abdomen much swelled—peritoneum inflamed throughout—a considerable quantity of sero-purulent fluid in that cavity, as well as layers of coagulable lymph over many of the abdominal viscera. The uterus was of the proper size, for the period *post partum;* but there was inflammation and a depôt of purulent matter between it and the rectum. "The blood in the venæ cavæ, subclavians, and right auricle of the heart, was reddish and serous, presenting striæ of whitish, yellowish, and grumous matters, mixed with fibrine." There were marks of inflammation, and also purulent matter in the sacro-iliac and ilio-pubic articulations, as in the first case. There was nothing else remarkable, except a few grumous particles, of a white or yellowish colour, in the branches of the hypogastric vein of the left side.

*Remarks.* We have laid these cases fully before our brethren, in order that they may see with what propriety the author, and some of our cotemporaries have brought them forward as proofs that *phlegmatia dolens* depends on inflammation of veins. In the first place, we deny that there are any proofs of the said inflammation. And in the second place we assert that, in neither of the three cases was there phlegmatia dolens at all. For the truth of this assertion, we appeal to all and every who have seen phlegmatia dolens—or who even have read the descriptions of it, as published in this country. There is not a single feature of correspondence between the diseases above related and phlegmatia dolens, except the *swelling*:—so that the author, and his copiers in this country, might just as well assert that a fractured femur was phlegmatia dolens, because, in both, the limb was swelled!

The diseases above related are interesting in themselves, as shewing fatal inflammations arising in certain parts after child-bearing; and the first and second cases are curious, as shewing the presence of purulent matter in the veins and absorbent vessels, with some strange alteration of the blood itself. But, as illustrations of the pathology or etiology of phlegmatia dolens, these cases are most exquisite specimens of ignorance and credulity.

---

14. *Obliteration of the Biliary Ducts.* Several curious and interesting cases of this kind are related in the "Archives," by the younger Andral, of which we shall give some account in this article. M. Andral thinks, and probably with reason, that many cases of icterus, attributed by practitioners to *spasm* of the biliary ducts, are, in reality, owing to inflammation of the membrane lining these conduits, and thus, more or less, completely stopping them up, either temporarily or permanently.

*Case* 1. A shoemaker, ætat. 35, was received into La Charité on the 8th November, 1821. Six days previously, after an excess in eating and drinking, he was suddenly seized with a violent pain at the epigastrium, a little to the right side. *9th.* He presented the following symptoms: viz. yellow tinge of the conjunctiva and of the whole surface of the body—obtuse pain in the right hypochondrium—a pyriform moveable tumour there, the extremity of which pointed under the false ribs, nearly as low as the line of the umbilicus—tongue natural—thirst moderate—appetite gone—stools scanty and colourless—pulse frequent—skin hot and dry. The tumour was considered to be the gall-bladder turgid with bile. *Leeches to the anus.* During the four following days the tumour increased in size, without any change in the other symptoms. On the 13th November, (eleventh day from the commencement of the attack) the patient was suddenly seized with an acute pain, which quickly spread over the whole of the abdomen. 14th. The pain continues, and is greatly exasperated by the slightest pressure—sufficiently indicating acute peritonitis. The face became pale and shrunk—anxiety great—pulse small and very quick—extremities cold. Died in the evening.

*Dissection.* The peritoneal cavity contained a good deal of purulent liquid of a yellowish colour. The internal surface of the duodenum was intensely red. Much difficulty was experienced in finding the entrance of the ductus communis choledochus, and, when found, it would scarcely admit the point of a fine probe. The whole of the canal was found to be greatly contracted, almost to obliteration, and its parietes thickened. The cystic and hepatic ducts, on the contrary, were very much distended, as was the gall-bladder itself. The hepatic duct, near the entrance of the cystic, was found to be burst, and through this opening, the bile had been extravasated into the abdomen, and there given rise to the peritoneal inflammation. There was no other organic change in any of the viscera.

*Case* 2. A man, 30 years of age, experienced, for two days, an acute pain in the right hypochondrium, after which he became jaundiced. When he entered LA CHARITE, the icterus and pain continued. A tumour of pyriform shape was detected below the ribs on the right side, which was moveable under the fingers. It appeared clearly to be the distended gall-bladder. The pulse was frequent—the skin hot—constipation obstinate. *Twenty leeches to the anus—lavemens—pediluvia.* During the three following days the tumour diminished—the jaundice was dissipated—the stools were re-established—and the patient was soon discharged cured.

*Case* 3. A man, 64 years of age, entered LA CHARITE in the latter part of December 1821. Three months previously, and without any known cause, he was seized with bilious vomitings, which continued some days. These having ceased, a diarrhœa came on, which lasted a month, and greatly reduced the patient. Towards the middle of September, the flux diminished, but the debility continued. The appetite was entirely gone—the food taken was digested with great difficulty—jaundice became established. Having entered the hospital, he presented the following phenomena:—viz. yellowish green jaundice—great emaciation—tongue nearly natural—anorexy complete—when a little food was taken it lay heavy at the pit of the stomach for several hours—stools scanty and unnatural—no tumour could be detected in the abdomen. Leeches to the epigastrium procured no relief—a blister was more serviceable. In about a fortnight, the patient's condition, as to his stomach, seemed a little ameliorated, but the icterus continued—and so did the debility and emaciation. One morning the patient felt something give way internally in the right hypochondrium, and a few minutes afterwards was seized with violent pain there, which spread over the whole of the abdomen. Acute peritonitis now immediately supervened, and was not arrested in its progress by numerous leeches to the abdomen. He died in the course of the night.

*Dissection.* A considerable quantity of greenish fluid was found in the abdomen. The gall-bladder was burst. The ductus communis and ductus cysticus were so contracted that the finest probe could not be passed along them. Their parietes were greatly thickened. The hepatic duct was much enlarged, and contained many biliary calculi. The substance of the liver was not altered. The mucous membrane of the stomach was thickened. Nothing else remarkable.

*Case* 4. A man, 50 years of age, entered La Charité in the beginning of December 1820, having been affected with icterus for seven months. He stated that he had never had any pain in the abdomen; but a swelling in that part had taken place during the last three months. The patient was now completely jaundiced, the face being of a yellowish green colour. The belly was enormously distended with water—the lower extremities slightly œdematous—appetite defective—the stools loose and entirely colourless—urine scanty and greenish—no pyrexia—thoracic organs apparently sound—not much inconvenience in respiration, and that produced by the fluid in the abdomen. This last was attributed to disease of the liver. *Ptisans, diuretics, mercurials, digitalis, &c.* During ten or twelve days, no change in the symptoms; but he suddenly expired about this period.

*Dissection.* The brain was soft and watery, but without any other lesion. Thoracic organs sound—peritoneal cavity filled with enormous quantity of water, of clear character, tinged a little of a citron colour—no appearance of inflammation in that membrane—liver remarkably small, and, as it were, wasted, and of an olive colour—it was otherwise of a natural texture—the primary biliary tubes considerably dilated, and filled with a grumous yellow matter, like the precipitate of bile by nitric acid—the ductus communis choledochus and ductus cysticus were transformed into a ligamentous cord, in which no cavity could be discovered by the most minute dissection—a biliary concretion in the gall-bladder, of the usual kind. The spleen greatly enlarged—the other digestive organs perfectly sound.

The foregoing cases may furnish some food for reflexion, both pathological and physiological. They prove, we think, that the bile is of some consequence in the animal economy—and that obstructions to its natural course into the intestines are not unattended by inconvenience or danger. From these obstructions to the course of *healthy bile*, we may form some idea of the effects of depraved—redundant—or defective secretion of the same fluid, on the digestive organs, and on the general health.

15. *Suppression of Urine.* Madame L—— had been affected with an ulcer on one of her legs for nearly 25 years. At length, she found the means of healing it, by an ointment procured from a charlatan. Shortly after the healing of the ulcer, she was seized with pains in the abdomen, inclination to vomit, and suppression of urine. M. Dupont, physician to the *Hospice de Gournay*, saw her on the fifth or sixth day. She had then some fever, moist skin, tongue dry and red, nausea, distention and pain of the abdomen. The catheter was introduced, but not more than an ounce of urine was found in the bladder. Fomentations and diluent diuretics were ordered. In three days more, M. Dupont visited the patient, and found her in nearly the same state. No secretion of urine. It was now that he learnt the circumstance of the healing of the old ulcer, and satisfied himself by examining the cicatrix. Suspecting a metastasis to the bladder, he applied a large blister to the site of the ulcer. The following day, a small quantity of urine was passed naturally. The proportion daily augmented, and in a few days, amounted to the usual quantity. All the other symptoms disappeared.—*Gazette de Santé.*

---

16. *Hæmatemesis fatal in* 14 *Hours.* M. Gaube (*Revue Med. Mars*, 1825) has related a case of this kind, the particulars of which are as follow. Madame ———, aged 40 years, of good constitution and embonpoint, complained to our author of a pain which she felt in the stomach immediately after taking some mutton broth. She thought she had swallowed a small bone of the mutton. As the pain was but trifling, and as deglutition was easy, our author made light of the complaint, and desired the patient to rest contented for the present. Seventeen days after this, viz. on the 11th December, 1824, our author was summoned in great haste to the patient, who was said to be dying of vomiting of blood. He found her senseless, without pulse, pale, extremities cold, having vomited up an enormous quantity of blood. Even in the above condition, the blood was coming up freely, partly in clots, partly florid. Frictions were applied to the extremities, and some cordial mixture was poured down the throat. The hæmatemesis was renewed every few minutes, preceded or succeeded by syncope, convulsions, and all the symptoms of approaching death. Various means were employed, but all to no purpose. She continued vomiting blood, with short intervals, till death put an end to the scene.

*Dissection.* All the great vessels, including the heart, were completely empty of blood. The lungs were sound. The œsophagus presented no trace of disease. The stomach was much distended with gas, and contained a large clot of blood. Its parietes shewed nothing particular, except two small patches of apparently recent inflammation. The parietes of the duodenum, on the other hand, were of a brownish black colour, especially on the internal surface, where the vessels were injected to the highest pitch.

We leave our readers to their own reflexions on this case. The reflexions of M. Gaube present nothing very interesting.

---

17. *Cardiac Aneurism in a Young Person.* Hypertrophy of the heart is certainly not very often seen in persons under 15 or 20 years of age; but we believe that both carditis and hypertrophy take place more frequently in young people than is generally supposed. M. Bricheteau has related a case in the Journal Complementaire, of which we shall take some notice here.

*Case.* Flore ——— H———, twelve years of age, not having yet menstruated, had enjoyed good health, and was of a very placid disposition till of late years. Latterly, she had complained of some difficulty of breathing, and of wandering pains in the chest. Her parents had led a most unhappy life, and the young lady was constantly witnessing their quarrels, and the ill-treatment of her mother. These scenes made a deep impression on her mind, and were even renewed in her dreams. Under these circumstances, she had an attack of rheumatism, which was cured, or supposed to be cured, by an empirical remedy. After this, she found difficulty in going up stairs, complained of palpitation, pain in the region of the heart, flushings of the face, and occasional sense of suffocation. When our author was consulted, she was in the following deplorable condition;—countenance pale, and indicative of great suffering—inability to lie down—breathing laborious—pulse quick and irregular, as were the cardiac pulsations, which could be felt over a considerable space—fixed pain in the region of the heart, aggravated in paroxysms, which generally came on in the evenings or in the night, accompanied by great palpitation and dry cough, with restlessness and anxiety. She was ordered to be bled from the arm, and revulsive pediluvia, as well as maniluvia, were directed. Other measures, of a palliative kind, were employed; but they merely gave temporary relief. She died on the 20th October, 1820.

*Dissection.* There were evidences of old inflammation, and some effusion in the right side of the chest. The heart was extremely voluminous. The pericardium was adherent to the heart throughout, and could not be separated from it. The vertical diameter of this organ measured 8 inches, (French) and the girt fourteen inches! The left chambers were augmented in size, and their parietes thickened—the right auricle and ventricle were also dilated, but their parietes were thinner than natural.

Another case is related by the same author of a young lady, 13 years of age, who, having lived in a damp situation, was affected with intermittent fever, for which she was removed to a dry and keen air. The ague disappeared, and was succeeded by affection of the breathing, palpitation, and, ultimately, cough. These symptoms continued to increase during two years, in spite of various means which were resorted to for their cure. Death at last put a period to her sufferings.

The comparative rarity of aneurismal affections of the heart, in children, may be easily accounted for by their comparative immunity from those various moral causes which so frequently produce the complaint in adult age. "De toutes les causes (says Corvisart) capables de produire les maladies du cœur, les plus puissantes, sans contredit, sont les affections morales." These moral causes, or agitations of mind, all, or almost all, seem to concentrate their effects on one of two points—the heart or the stomach. Hence, diseases of the circulating system and of the digestive organs, with all their various concomitants and consequences.

---

18. *Pericarditis, and other Phlegmasiæ, from a Moral Cause.* (*Archives Generales, Fevrier*, 1825.) A young ecclesiastic, 20 years of age, much given to intellectual pursuits, was subject to sudden and considerable bleedings from the nose, and to no other malady. On the first day of October, 1822, he experienced some disappointments, from which time he appeared depressed in spirits, and much altered in his countenance. His eyes seemed to himself to be sometimes covered as with a mist—profuse hæmorrhages from the nose several times in the 24 hours—insomnium obstinate—afterwards, violent cephalalgia—progressive loss of sight—sense of tightness in the anterior part of the chest, preventing the recumbent posture on the back—anxiety—cough, and frothy sanguineous expectoration. 8th October, 6th day of these symptoms, 15 leeches were applied to the chest—rigid abstinence—diluents. Towards mid-day, the patient found himself worse, although the leech-bites were bleeding freely. Dr. Bidois now saw the patient, and found him sitting up and leaning forward in his bed—his face puffy—lips and conjunctivæ exceedingly pale—sight much enfeebled—vertigo—breathing laborious—sense of fulness and heat in the chest—cough troublesome, with frothy red expectoration—great sense of suffocation—constant restlessness—inability to lie down—strong præcordial pulsations—pulse at the wrist full, hard, and corresponding with the action of the heart—tongue pale, and slightly yellow at the root—extremities cold. Bled to 12 ounces from the arm, and the leech-bites encouraged. *Evening.* Great amelioration of symptoms from the bleeding—especially in the freedom of the chest, and the diminution of the redness in the expectoration, and strength and frequency of the pulse. 9th and 10th. The amelioration continued—the patient could lie down on the right side, and get some hours' sleep. Still there was tightness in the chest—and, although the other symptoms were abated, the præcordial pulsations were extremely violent. The swelling of the face has augmented; and the same obtains in the hands and feet. The same remedies, *but no further sanguineous evacuation.* After some days the patient seemed much better—could walk about his room—and receive his friends. On the 19th of October, the patient, yet convalescent, stood at the window of a very small room for several hours, talking much, and that day eating and drinking more than was prudent. In the evening, he had severe cephalalgia, and return of the pectoral symptoms before enumerated, which became progressively aggravated towards midnight. At this time, the anxiety and oppression at the chest were intolerable. He was therefore bled to ten ounces. 20th Oct. The relief from the bleeding was less marked than on the former occasion. The blood was buffed, but the buff was not very thick. In the evening there was epistaxis. *Pediluvia—juleps, with syrup of poppies.* Night very restless. 21st. The epigastrium very tender on pressure—complains that the drink makes him sick—the thoracic symptoms still continue. The contractions of the heart make the whole thorax vibrate. 22nd. There being no abatement of the symptoms, ten ounces of blood were abstracted—cataplasms, &c.—fomentation to the epigastrium, which was become very painful. The cataplasms could not be borne on the epigastrium—bilious vomitings this morning, preceded by a sense of burning heat at the cardiac orifice of the stomach. 23d and 24th. Constant vomiting, day and night, of bilious and greenish matters, streaked occasionally with a little blood, and still accompanied by burning heat at the pit of the stomach. Things went on from bad to worse. The thoracic affections were, in some degree, veiled, as it were, by the intensity of the gastric symptoms. He died on the 26th October.

*Dissection, 24 hours after death. Abdomen.*—About a pint and a half of yellow serum in the cavity of the peritoneum, of heavy odour, and containing gelatinous flocculi. The stomach was much contracted, of a reddish colour externally, and its vessels very much injected. Internally it contained but a small quantity of ropy fluid. Its parietes were thickened—its mucous lining of an orange colour from inflammation, especially about the cardiac orifice, the redness gradually diminishing as the pyloric orifice was approached. In the duodenum, the redness and inflammation again appeared in a very high degree, and was prolonged, with more or less intensity, into the small intestines. The internal surface of the œsophagus of a vinous red colour, especially its lower half—liver very much enlarged. *Thorax.*—In the bags of the pleura, on both sides, there were several ounces of yellowish serum, with gelatinous flocculi, similar to that found in the cavity of the peritoneum. The pleural linings not sensibly affected themselves. The lungs were sound, but slightly infiltrated. The mucous membrane of the trachea and bronchia of a reddish violet colour. *Heart.*—Some ounces of serum in the cavity of the pericardium—the membrane itself, both its loose and reflected portions, of a yellowish colour, rough, thickened, and unequal—in short, intensely inflamed. The size of the heart was considerably greater than natural, for the stature of the man. The left ventricle was thickened in parietes and enlarged in cavity.

M. Bouillaud has made some reflexions on the above case. He comments on the neglect of auscultation, whereby the nature of the thoracic malady was not appreciated. In this we agree with M. B. but then we say that no practical man in this country would have been led into the error of M. Bidois, whether he had a stethoscope or not. The symptoms indicated dangerous inflammation in the chest, and these were to be combated by active depletion, *whatever was the actual seat of the phlogosis.* There is scarcely an apothecary's apprentice in this country—certainly not a dresser at an hospital, who would not have saved this young clergyman's life by copious bloodletting, even from the first blush of the symptoms which were presented. Who, in his senses, on seeing a young man unable to lie down in bed—coughing—spitting up frothy, bloody expectoration—with sense of suffocation, and hard, full pulse, would not have instantly unsheathed his lancet, and bled him till the symptoms were relieved or syncope intervened?—None in this country. M. Bouillaud says—"que nous aimons à placer M. Bidois parmi les bons observateurs." His friend may be a good *observer*—but we maintain that he is a very bad *practitioner*.

